# Effectiveness of porous silicon nanoparticle treatment at inhibiting the migration of a heterogeneous glioma cell population

**DOI:** 10.1186/s12951-021-00798-4

**Published:** 2021-02-26

**Authors:** Youssef Abdalla, Meihua Luo, Ermei Mäkilä, Bryan W. Day, Nicolas H. Voelcker, Wing Yin Tong

**Affiliations:** 1 School of Pharmacy, University College London, 29-39 Brunswick Square, London, WC1N 1AX UK; 2 Drug Delivery, Disposition and Dynamics, Monash Institute of Pharmaceutics Science, Monash University, Parkville Campus, 381 Royal Parade, Parkville, VIC 3052 Australia; 3 Department of Biomedical Engineering, The Chinese University of Hong Kong, Shatin, New Territories Hong Kong; 4 Industrial Physics Laboratory, Department of Physics and Astronomy, University of Turku, Turku, Finland; 5 Sid Faithfull Brain Cancer Laboratory, QIMR Berghofer Medical Research Institute, Brisbane, QLD Australia; 6 Commonwealth Scientific and Industrial Research Organization (CSIRO), Clayton, VIC Australia; 7 Melbourne Centre for Nanofabrication, Victorian Node of the Australian National Fabrication Facility, Clayton, VIC Australia; 8 Department of Materials Science and Engineering, Monash University, Clayton, VIC Australia; 9 Leibniz Institut für Neue Materialien (INM), Campus D2 2, 66123 Saarbrücken, Germany

**Keywords:** Transferrin, Silicon nanoparticles, Glioma, Glioblastoma, Cell migration, aquaporin 9, Cancer stem cells

## Abstract

**Background:**

Approximately 80% of brain tumours are gliomas. Despite treatment, patient mortality remains high due to local metastasis and relapse. It has been shown that transferrin-functionalised porous silicon nanoparticles (Tf@pSiNPs) can inhibit the migration of U87 glioma cells. However, the underlying mechanisms and the effect of glioma cell heterogeneity, which is a hallmark of the disease, on the efficacy of Tf@pSiNPs remains to be addressed.

**Results:**

Here, we observed that Tf@pSiNPs inhibited heterogeneous patient-derived glioma cells’ (WK1) migration across small perforations (3 μm) by approximately 30%. A phenotypical characterisation of the migrated subpopulations revealed that the majority of them were nestin and fibroblast growth factor receptor 1 positive, an indication of their cancer stem cell origin. The treatment did not inhibit cell migration across large perforations (8 μm), nor cytoskeleton formation. This is in agreement with our previous observations that cellular-volume regulation is a mediator of Tf@pSiNPs’ cell migration inhibition. Since aquaporin 9 (AQP9) is closely linked to cellular-volume regulation, and is highly expressed in glioma, the effect of AQP9 expression on WK1 migration was investigated. We showed that WK1 migration is correlated to the differential expression patterns of AQP9. However, AQP9-silencing did not affect WK1 cell migration across perforations, nor the efficacy of cell migration inhibition mediated by Tf@pSiNPs, suggesting that AQP9 is not a mediator of the inhibition.

**Conclusion:**

This in vitro investigation highlights the unique therapeutic potentials of Tf@pSiNPs against glioma cell migration and indicates further optimisations that are required to maximise its therapeutic efficacies.

**Graphic Abstract:**

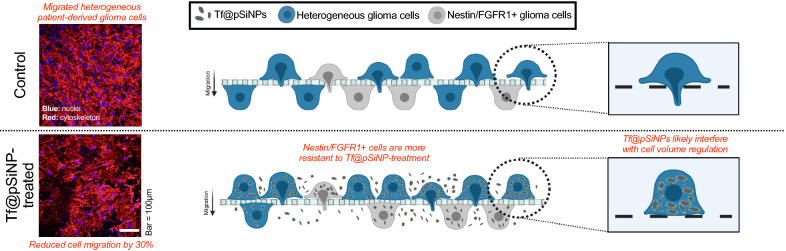

## Background

Brain tumours are notoriously refractory to therapy. Approximately 80% of brain tumours are gliomas, primary tumours that arise from neuroglial stem or progenitor cells [1]. Glioblastoma (GBM) is the most common and aggressive form of the disease [2]. GBM is highly malignant and resistant to therapy [3]. Standard of care typically involves maximum resection followed by radiotherapy and adjuvant temozolomide (TMZ) [4]. However, patient survival is approximately 15 months from diagnosis due to a high rate of relapse [1]. This often occurs as a result of incomplete tumour resection, owing to a diffuse tumour boundary and extensive tumour heterogeneity. The diffuse tumour boundary is created by a highly motile subpopulation of glioma cells [5]. At the edge of the tumour, migrating cells undergo biochemical changes, such as increased expression of integrins [6] and matrix metalloproteinases (MMP) [7]. As a result, some of these cell subpopulations exhibit a significantly lower proliferation rate then the cells at the tumour core [8]; this is known as the ‘Go or Grow’ mechanism. It is clinically evident that despite 99% resection of the glioma mass, relapse occurs within 6 months [9]. This is partly due to the migrated cells left behind after incomplete resection readopting a proliferation phenotype and reforming a tumour mass [5]. Most therapies to-date treat proliferative cancer cells through inhibition of DNA replication [9]. The absence of measures to inhibit glioma cell migration thus presents a major barrier to realise a complete treatment of GBM [5].

We previously demonstrated that transferrin-functionalised porous silicon nanoparticles (Tf@pSiNPs) inhibit glioma cell migration by approximately 40% using the immortalised U87 glioma cell line model [10]. Porous silicon nanoparticles (pSiNPs) have been studied extensively as they are both biocompatible and biodegradable. The degradation product of pSiNPs is orthosilicic acid, which is non-toxic [11, 12]. In addition, they have a high surface area to volume ratio, allowing high drug loading [13]; they also have pores of homogenous size which can be altered to tailor drug loading and release [14] and a silanol-containing surface, allowing functionalisation for targeted delivery [13, 15] and/or controlled drug release [16]. The functionalisation of pSiNPs with transferrin targets the transferrin receptor, which is overexpressed on gliomas [17], resulting in selective uptake of Tf@pSiNPs via clathrin receptor-mediated endocytosis [18]. Transferrin functionalisation has been found to increase the rate and extent of nanoparticle-uptake by glioma cells [18], and to allow traversing across in vitro models of the blood brain barrier (BBB) [18, 19].

Cellular heterogeneity has been identified as a hallmark of GBM [20]. Within a tumour, there is a hierarchal organisation of cancer cells, including cancer stem cells (CSCs) [21]. CSCs are cells in a tumour with the capacity to migrate, self-renew, and generate the entire tumour cell population [22]. They are associated with tumour initiation, metastasis and relapse [23] and are more resistant to conventional treatments than the tumour bulk [24, 25]. This resistance is acquired through quiescence, increased ATP-binding cassette transporter-expression [25] and the utilisation of DNA repair mechanisms [24]. One such example is O^6^-methylguanine-deoxyribonucleic acid methyltransferase (MGMT), which dealkylates guanine and hence confers resistance to alkylating agents, such as temozolomide [25]. Various GBM cell subpopulations are characterised by the presence of specific membrane proteins. For example nestin, a class VI intermediate filament protein found in the cell cytoplasm, and a CSC marker [26, 27]; and fibroblast growth factor receptor 1 (FGFR1), a tyrosine kinase receptor [28] which enhances cancer cell proliferation and migration [29].

A more thorough understanding of the mechanism of Tf@pSiNPs’ migratory inhibition would allow treatment optimisation. It is established that during local metastasis pronounced changes in cell volume (30–35%) are central to glioma cell navigation through the brain parenchyma [30, 31]. We deduced previously that Tf@pSiNPs attenuate cell migration by inhibiting cell volume reduction in response to osmotic pressure [10]. However, how exposure to Tf@pSiNPs is translated into inhibition of cell volume regulation remains elusive. One speculation is the inhibition of cytoplasmic water removal by inhibiting aquaporin (AQP) functions upon treatment with Tf@pSiNPs. Physiologically, AQPs increase the plasma permeability to water by 5–50 times, allowing rapid cell volume changes [30]. The role of aquaporins in cell migration, and the invasiveness of many cancers is well established [32]. Noteworthily, aquaporin 9 (AQP9), out of all AQPs, mediates the most orthosilicic acid transfer across the cell membrane in human cells [33]. However, whether AQP9 has a role in glioma cell volume regulation, migration, and mediating the migratory inhibition caused by Tf@pSiNPs has never been studied.

U87 cells are long term, immortalised GBM cells. Whilst these cells are highly proliferative and easily cultured, they do not maintain a stem cell-like phenotype. The Q-Cell WK1 primary cell line model was generated from a tumour specimen resected from a 77-year-old male with primary isocitrate dehydrogenase 1 (IDH1) wild-type, MGMT unmethylated GBM. WK1 cells have been characterised in great detail at the genetic, molecular and proteomic level and are predominantly of mesenchymal subtype origin [34, 35]. WK1 cells have been shown to form orthotopic tumours in immunocompromised mice and are readily cultured under serum-free glioma neural stem (GNS) cell conditions [36]. While the U87 cell line is good for identifying initial responses, the early passage WK1 cell line model is needed to confirm our positive findings, as this better represents tumour stemness and intratumoural heterogeneity.

Here, we used WK1 cells to evaluate the anti-migratory effect of Tf@pSiNPs. We observed that Tf@pSiNPs significantly inhibited WK1 cell migration across 3 μm, but not 8 μm perforations. In alignment with our observation with U87 cells, Tf@pSiNPs did not alter actin filament formation in WK1 cells. This indicates that the mechanism of cell migration inhibition mediated by Tf@pSiNPs on a heterogenic GBM cell population is dependent on cellular volume. Although AQP9 is closely related to cell volume regulation and silicon ion exchange, we excluded the role of AQP9 in mediating the cell migration inhibition induced by Tf@pSiNPs as AQP9 silencing did not affect cell migration. Since silicon ion uptake alone did not affect cell migration across confinements, we confirmed that the contribution of pSiNPs degradation on anti-migratory effect is negligible. Despite tumour heterogeneity being a root cause of treatment failure, Tf@pSiNPs inhibited WK1 cell migration by approximately 30%. Among the subpopulations that were able to migrate across 3 μm pores, a majority of these cells were shown to be nestin and/or FGFR1 positive, indicating a potential CSC or de-differentiated origin. This in vitro evaluation highlights the unique therapeutic potential of Tf@pSiNPs against GBM cell migration, and the therapeutic anti-migratory effect of Tf@pSiNPs, which could be further optimised by incorporating a more effective CSC-targeting strategy.

## Results

We previously found that Tf@pSiNPs inhibit the migration of U87 glioma cells through 3 μm channels in microfluidic-based migration chips [10]. This exciting discovery prompted us to further verify the effect in a more physiologically-relevant, heterogeneous, patient-derived glioma cell line (WK1), which represents a major hurdle in the development of GBM treatments [37], and to better clarify the mechanism of action.

**Fig. 1:**
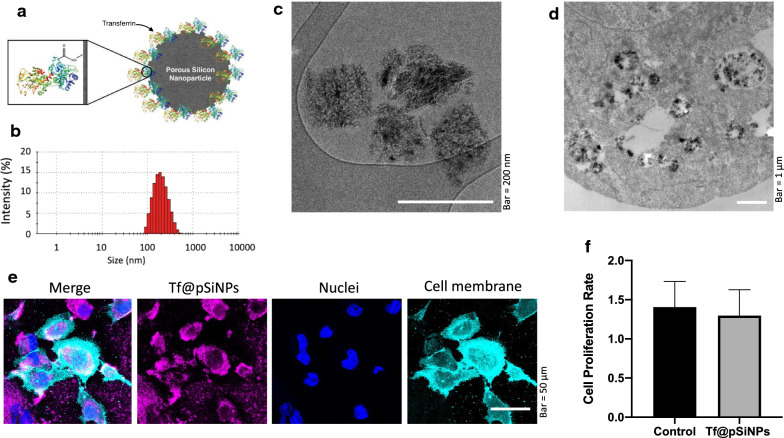
Tf@pSiNPs characterisation and uptake. **a** Representation of Tf@pSiNPs (not to scale). Transferrin structure obtained from Protein Data Bank [38]. **b** Hydrodynamic particle size distribution of Tf@pSiNPs, as indicate by DLS. **c** Cryo-TEM image of Tf@pSiNPs **d** Cryo-TEM image of Tf@pSiNPs in glioma cells. **e** Confocal microscopy imaging to show Tf@pSiNPs’ uptake by WK1 cells. Cyanine5 (magenta), Vybrant (cyan) and Hoechst 33,342 (blue) staining allowed visualisation of Tf@pSiNPs, cell membrane and nuclei, respectively. **f** WK1 cell proliferation rate following treatment with Tf@pSiNPs, quantified as the ratio between the number of cells 24 and 48 h post-seeding (data presented as mean ± 1 SD, n = 3), no significant difference was found following treatment with Tf@pSiNPs (Student’s t-test). *Tf@pSiNPs* transferrin-functionalised porous silicon nanoparticles, *DLS* dynamic light scattering, *TEM* transmission electron microscope

Tf@pSiNPs, shown in Fig. 1a, were produced by electrochemical anodisation of p-type boron-doped Si wafers, followed by transferrin functionalisation. The hydrodynamic particle size distribution and zeta potential were characterised using dynamic light scattering (DLS). The average particle size was determined to be 182 ± 1 nm (Fig. 1b), with a narrow size distribution as indicated by a polydispersity index of 0.10 ± 0.02. This was further confirmed by the consistent size and shape observed under cryo-transmission electron microscope imaging (Fig. 1c, d). The zeta potential was found to be − 9 ± 1 mV. This data highlights the consistency of the physical properties of Tf@pSiNPs and their colloidal stability. Uptake of Tf@pSiNPs by WK1 cells was also assessed using confocal microscopy (Fig. 1e). Cell proliferation rate was measured following treatment with Tf@pSiNPs. No significant difference was found between the proliferation rate of WK1 cells treated with Tf@pSiNPs and controls (Fig. 1f). This indicates that the internalisation of Tf@pSiNPs is unlikely to be toxic to WK1, in agreement with our previous observation that treatment with Tf@pSiNPs does not affect the ATP content of cells [18]. Therefore, toxicity is unlikely to play a role in the migration inhibition.

**Fig. 2:**
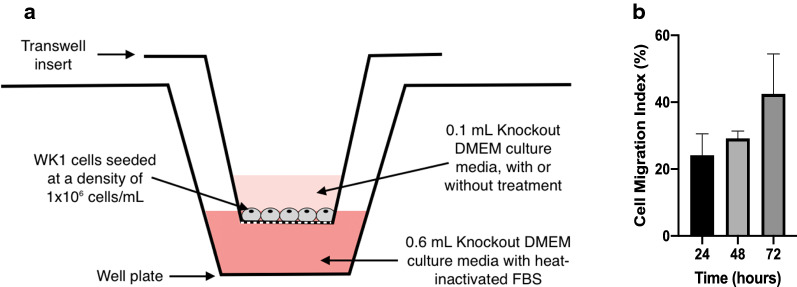
Identifying control cell migration. **a** Cell migration assay apparatus (not to scale). **b** Quantification of WK1 cell migration at 24, 48 and 72 h expressed as the cell migration index (data presented as mean ± 1 standard deviation, n = 3)

Since there was no precedent to this study about the migration rate of WK1 cells across narrow confinements, we first challenged the migration of WK1 cells in the absence of any treatment over a 72 h time frame in transwell inserts with 3 µm perforations (Fig. 2a). Expectedly, successful migration (cell migration index) increased non-linearly over the 72 h. The cell migration index (CMI), which is the ratio between the number of migrated cells and the total number of cells, reached approximately 43% (Fig. 2b). It was determined that approximately 29%, a considerable proportion of the cells, had migrated 48 h after seeding. We thus chose 48 h post-seeding as a point to compare CMI for the rest of the study.

**Fig. 3:**
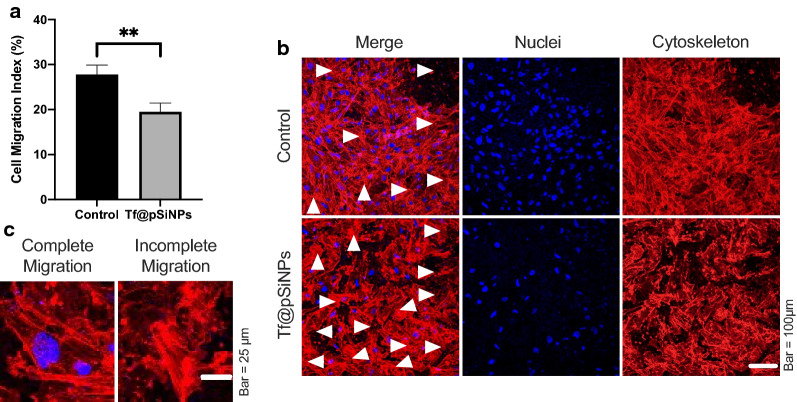
Tf@pSiNPs significantly reduced WK1 cell migration. **a** Quantification of cell migration at 48 h (data presented as mean ± 1 standard deviation, ** indicates p < 0.01, Student’s t-test, n = 3). **b** Image of cells on the underside of the transwell membrane; white arrows mark areas where cells have not completely cleared the perforation. **c** Magnified image of cells on the underside of the transwell membrane, showing examples of cells that completed migration across the perforations, and those that did not. Phalloidin (red) and Hoechst 33,342 (blue) staining allowed visualisation of the cytoskeleton and nuclei, respectively. *Tf@pSiNPs* transferrin-functionalised porous silicon nanoparticles

When comparing the CMI of cells treated with Tf@pSiNPs to control cells, we observed that Tf@pSiNPs reduced cellular migration across 3 μm confinements by roughly 30% (Fig. 3a). It is interesting that although many nuclei of WK1 cells treated with Tf@pSiNPs had not completely cleared the perforation, the protrusions of those cells were observable using cytoskeleton staining (Fig. 3b, Arrows and Fig. 3c).

**Fig. 4:**
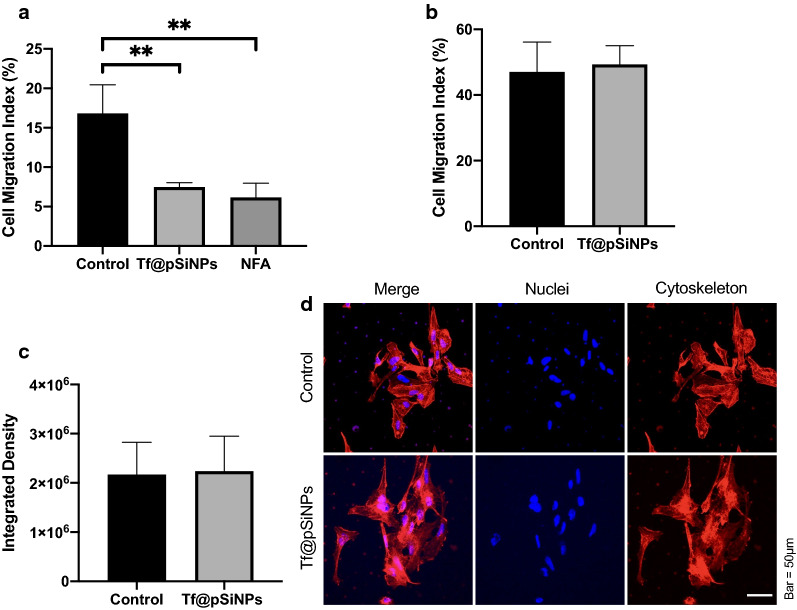
WK1 cell migration across 3 μm perforations over 48 h with different treatments. **a** Quantification of cell migration, across 3 μm perforations, following Tf@pSiNPs- or NFA-treatment (data presented as mean ± 1 SD, ** indicates p < 0.01, one-way analysis of variance, n = 3). **b** Quantification of WK1 cell migration across 8 μm perforations following treatment with Tf@pSiNPs (data presented as mean ± 1 SD, n = 3); no significant difference in migration was observed (Student’s t-test). **c** Quantification of the intensity of cytoskeletal staining, following treatment with Tf@pSiNPs (data presented as mean ± 1 SD, n = 3) no significant difference was observed (Student’s t-test). **d** Representative image of the cytoskeletal structure of cells under nanoparticle treatment. Phalloidin (red) and Hoechst 33,342 (blue) staining allowed visualisation of the cytoskeleton and nuclei, respectively. *Tf@pSiNPs* transferrin-functionalised porous silicon nanoparticles, *NFA* niflumic acid, *SD* standard deviation

The promising result of migratory inhibition on heterogenic WK1 cells prompted us to further explore the mode of action of the inhibition. We previously showed that cell volume reduction was required for U87 cell migration across physical confinements [10]. To show that patient-derived glioma cells require the same criteria, we studied the effect of niflumic acid (NFA) on CMI. NFA is a calcium-activated chloride channel inhibitor [39], it inhibits cell volume regulation [40], and was therefore used as a positive control. We observed that both NFA and Tf@pSiNPs significantly inhibit cell migration (Fig. 4a), but no significant difference in CMI was observed between Tf@pSiNPs- and NFA-treatment, suggesting that they are both of similar efficacy at inhibiting migration through 3 μm transwells, as was previously observed with U87 cells [10]. To further establish whether Tf@pSiNPs inhibited cell migration by disrupting volume regulation, WK1 cell migration through 8 μm perforations, which posed less confinement, was assessed. We found that WK1 cells treated with Tf@pSiNPs were able to migrate across 8 μm transwell inserts, and no significant difference in CMI was observed, compared to control cells (Fig. 4b). In addition, the nanoparticles had no effect on cytoskeletal formation, with no changes observed in either the intensity of cytoskeletal staining (Fig. 4c) or the observed cytoskeletal structures (Fig. 4d), following treatment with Tf@pSiNPs. This agrees with what we had previously hypothesised, that Tf@pSiNPs prevent the cell volume changes which mediate cell migration [10]. Since most of the rapid cytosol volume changes occur through the action of AQPs [30], we speculated that Tf@pSiNPs modulated cell volume changes by acting on AQPs.

**Fig. 5:**
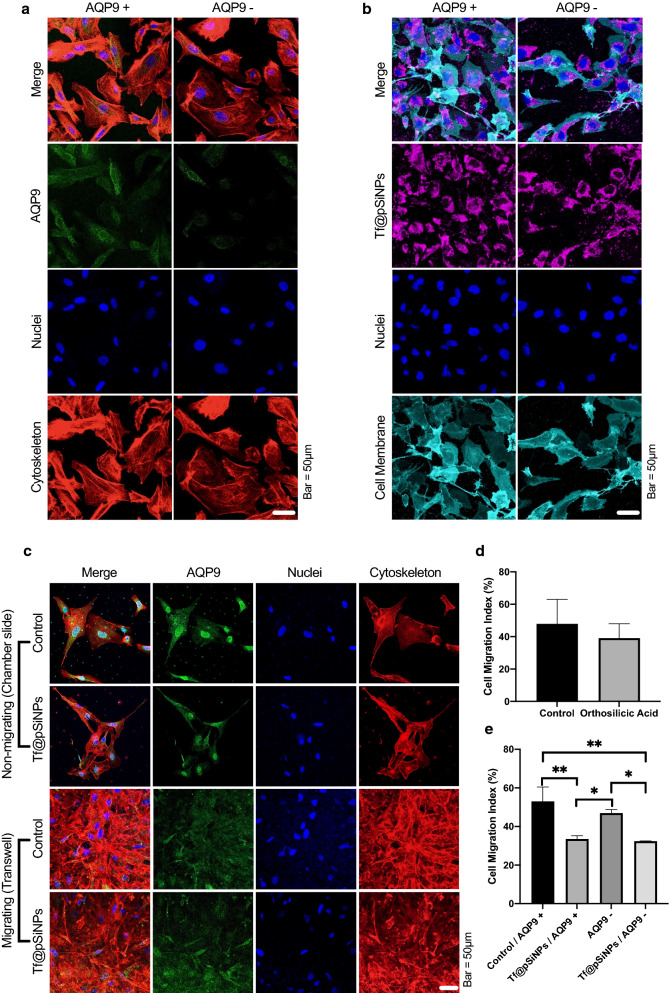
AQP9 was not involved in Tf@pSiNPs’ mode of action. **a** AQP9 silencing confirmation following transfection. **b** Image of cells in a chamber slide to evaluate the uptake of Tf@pSiNPs after AQP9 silencing. **c** Image of cells in a chamber slide or the underside of a transwell, to identify the expression of AQP9 in non-migrating and migrating cells, respectively, with and without Tf@pSiNPs. **d** Quantification of cell migration following orthosilicic acid-treatment (data presented as mean ± 1 SD, n = 3) no significant difference was observed (Student’s t-test). **e** Quantification of cell migration at 48 h (data presented as mean ± 1 SD, * indicates p < 0.05, ** indicates p < 0.01, one-way analysis of variance, n = 3). Phalloidin (red), Alexa 488 (green), Cyanine5 (magenta), Vybrant (cyan) and Hoechst 33,342 (blue) staining allowed visualisation of the cytoskeleton, AQP9, Tf@pSiNPs, cell membrane and nuclei, respectively. *Tf@pSiNPs* transferrin-functionalised porous silicon nanoparticle. *AQP9* aquaporin9, *siRNA* small interfering ribonucleic acid

AQP9 is overexpressed in gliomas [32]. Apart from its function as a water transporter, it is largely involved in silicon transfer [33]. This prompted us to verify the involvement of AQP9 in WK1 migration and the cell migration inhibition mediated by Tf@pSiNPs. The silencing of AQP9 in WK1 was achieved by siRNA transfection and confirmed by immunofluorescence imaging (Fig. 5a).

We hypothesised that AQP9 may be involved in the uptake of Tf@pSiNPs; therefore, we studied the effect of AQP9 silencing on the uptake of Tf@pSiNPs in AQP9 + and AQP9- WK1 cells. Notably, no difference in the uptake of Tf@pSiNPs was observed between the two groups (Fig. 5b), indicating that AQP silencing did not alter the internalisation of Tf@pSiNPs.

To identify any changes in AQP9 localisation during migration, in the presence and absence of nanoparticles, the expression of AQP9 in both migrating and non-migrating cells was investigated. Interestingly, it was observed that AQP9 localised predominantly at the nuclear membrane of non-migrating cells, whereas AQP9 expression appeared more diffuse and cytoplasmic in migrating cells (Fig. 5c). However, the observed AQP9 expression pattern was unaffected by Tf@pSiNPs.

pSiNPs degrade into orthosilicic acid in biological fluid [11]. Apart from water transport, AQP9 was reported to facilitate ionic silicon exchange across the cell membrane. Therefore, we hypothesised that orthosilicic acid may have contributed to the observed anti-migratory effect, if AQP9 was involved. It was confirmed by inductively coupled plasma mass spectrometry (ICPMS) that 140 µg of Si ions were found in a pool of 3.5 × 10^5^ cells treated with pSiNPs. To isolate and maximise the potential anti-migratory effect of orthosilicic acid exposure on WK1 cells, cells were treated with orthosilicic acid at its maximum solubility (192 µg/ml) [41]. It was observed that the CMI of WK1 cells treated with orthosilicic acid was not significantly different from untreated control cells (Fig. 5d). This indicates that the inhibition of cell migration observed was due to the nanoparticles themselves and not their degradation products.

Migration assays were then carried out to probe the effect of AQP9 silencing on cell migration. While Tf@pSiNPs still inhibited the migration of WK1 cells, AQP9 silencing in WK1 alone had no significant effect on reducing cell migration (Fig. 5e). Notably, the CMI of AQP9 + and AQP9- WK1 cells treated with Tf@pSiNPs was not significantly different. This indicated that AQ9 function is not fundamental to WK1 cell migration across confinements, nor is it functional to the efficacy of Tf@pSiNPs.

**Fig. 6:**
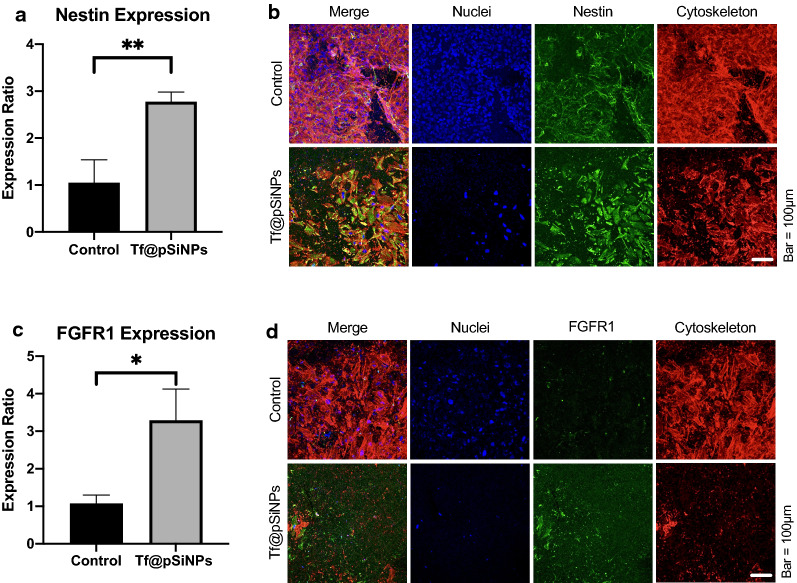
A greater proportion of WK1 cells migrating after treatment with Tf@pSiNPs express Nestin/ FGFR1. **a** Ratio of Nestin expression on the underside of the membrane to the top side (data presented as mean ± 1 SD, ** indicates p < 0.01, Student’s t-test, n = 3). **b** Image of cells at the underside of the transwell membrane, showing cells that have successfully migrated, and nestin-expression in those cells. Phalloidin (red), Alexa 488 (green) and Hoechst 33,342 (blue) staining allowed visualisation of the cytoskeleton, nestin and nuclei, respectively. **c** Ratio of FGFR1-expression on the underside of the membrane to the top side (data presented as mean ± 1 SD, * indicates p < 0.05, Student’s t-test, n = 3). **D** Image of cells at the underside of the transwell membrane, showing cells that have successfully migrated, and FGFR1 expression on those cells. Phalloidin (red), Alexa 488 (green) and Hoechst 33,342 (blue) staining allowed visualisation of the cytoskeleton, FGFR1 and nuclei, respectively. *Tf@pSiNPs* transferrin-functionalised porous silicon nanoparticles, *FGFR1* fibroblast growth factor receptor 1, *SD* standard deviation

Gliomas contain largely heterogenous cell subpopulations [42]; therefore, the efficacy of Tf@pSiNPs depends on the cumulative effect on each cancer cell subpopulation. We demonstrated that the overall migratory inhibition of WK1 cells was approximately 30%. Revealing the phenotypic characteristics of migrated cells treated with Tf@pSiNPs will provide specific insights into both glioma invasiveness and possibilities in optimising the effectiveness of Tf@pSiNPs. It has been proposed that the existence of CSCs in gliomas plays a major role in driving disease progression and recurrence. We therefore performed migration assays and utilised immunofluorescence staining to identify and compare glioma CSC-specific markers. The cells on both sides of the transwell membrane were analysed following treatment with Tf@pSiNPs. It was observed that a significantly higher proportion of migrating cells treated with Tf@pSiNPs expressed nestin compared to the control, suggesting that these cells could be of glioma CSC origin (Fig. 6a, b). Among those cells, we also demonstrated that a significantly higher proportion of them were FGFR1 positive (Fig. 6c, d). FGFR1 is a tyrosine kinase receptor [28] which enhances cancer cell migration [29]. This may provide insight into the reason why these cells were insensitive to Tf@pSiNPs.

## Discussion

Although extensive brain infiltration is a hallmark of GBM and is associated with high mortality, research into approaches to counter this strong migratory effect is scarce. Despite the mainstream use of pSiNPs for drug loading, such as doxorubicin [18], we found that blank pSiNPs functionalised with transferrin to aid glioma-specific cell entry, are not cytotoxic [18], and can be effective at disrupting U87 cell migration across narrow physical confinements [10]. Whilst this encouraging result implicated promise of a novel treatment approach, a deeper understanding of the inhibitory mechanism and evaluation of its effectiveness was needed.

To extend our previous studies, we assessed the anti-migratory effect of Tf@pSiNPs on a patient derived glioma cell model (WK1). Unlike immortalised glioma cells such as U87, these primary, early passage GBM cells maintain a stem cell-like phenotype and better retain the pathological characteristics of tumour heterogeneity [43]. Cell migration models conventionally gauge cells migrating across transwells with perforated membranes of a particular pore size. Watkins et al*.* [31] found that the smallest pore size D54-MG and U251-MG glioma cells can traverse through is 5 μm. In our recent study, we observed that U87 cells can traverse across 3 μm microchannels of our specially made migration chip in 24 h [10]. However, our pilot study showed that WK1 cells required more than 3 days to complete migration in the same system (data not shown). Since there is no technical advantage in using migration chips for such long-term study, we decided to assess the migration of WK1 cells using a transwell model with membranes containing 3 μm perforations.

The striking difference in migration speed across the same physical confinement between U87 and patient derived WK1 cells intrigued and prompted us to explore the efficacy of Tf@pSiNPs at inhibiting the migration of WK1 cells. Tf@pSiNPs were non-cytotoxic and significantly reduced the migration of WK1 cells by approximately 30%. This is less than what was previously observed with U87 cells, which was approximately 40% migratory inhibition [10]. There are two possible reasons for the interesting difference in migratory inhibition observed. Firstly, the mechanism of migratory inhibition of Tf@pSiNPs in U87 cells may be fundamentally different from WK1 cells. Secondly, since the primary cell line has greater cell heterogeneity, as compared to lab acclimatised U87, subpopulations of cells that are less susceptible to the effect of Tf@pSiNPs may exist [44]. In particular, this 10% difference may be attributed to the presence of a CSC population in WK1 cells, which is less susceptible to treatment with Tf@pSiNPs (Fig. 6) and is established as being more invasive than non-CSCs [45–48]. For example, Volovetz et al*.* [46] found that glioma stem cells showed 2–5 times greater cell motility when compared to non-CSCs. Our observation is further consolidated by previous investigations showing that on average glioma stem cells constitute approximately 10–13% of primary glioma cells [26, 49, 50].

Previous studies have shown that nanoparticles can inhibit cell migration, although through different mechanisms of action. Ali et al*.* [51] found that gold nanoparticles can inhibit cell migration of ovarian cancer cells in a scratch migration assay by increasing nuclear stiffness, both directly, and indirectly through increased expression of lamin A/C around the nuclear membrane. This is unlikely to be the case with Tf@pSiNPs as they did not show localisation around the nuclear membrane, but instead were found more diffusely throughout the cytoplasm, in both U87 [10] and WK1 cells (Fig. 5b). Gold nanoparticles function differently to gold nanorods, which were investigated by Zhou et al*.* [52]. They found that gold nanorods coated with BSA impaired adenosine triphosphate (ATP) synthesis and hence filamentous-actin cytoskeletal assembly, which reduced the migration of breast cancer, prostate cancer and melanoma cells [52]. Tay et al*.* [53] investigated the effect of nanoceramics on wound healing using silicon dioxide, titanium dioxide and hydroxyapatite nanoparticles. They found that the nanoparticles reduced wound healing through the disruption of microtubule assembly. Similar migration levels were observed with all three nanoparticle treatments, suggesting it could be due to a similar composition of proteins surrounding the nanoparticles. The proteins increase the strength of substrate adhesion and induce fibrous microtubule dissolution, limiting migration [53].

We found that Tf@pSiNPs do not affect WK1 cell proliferation. Furthermore, in our recent studies utilising Tf@pSiNPs, we observed that ATP production and cell viability of U87 cells and other brain cells were not affected by the internalisation of Tf@pSiNPs [18]. Therefore, we deduced that cell toxicity did not play a role in the observed inhibition. On the U87 model, we observed that Tf@pSiNPs inhibited cells migrating across confinements, while the unrestricted migration in a scratch migration assay was unaffected [10]. Since cytoskeletal-disruption inevitably affects both unrestricted migration and cell spreading [54], it is unlikely that Tf@pSiNPs inhibit the migration of U87 cells through the disruption of the cytoskeleton. Indeed, the effect of Tf@pSiNPs on WK1 cells’ migration resembles our recent observations in U87, whereby the inhibition was observed for cells migrating across 3 μm perforations but not across 8 μm, with no effect on the WK1 cells’ cytoskeletal formation. We thus believe that the migratory inhibition induced by Tf@pSiNPs in U87 and WK1 cells were based on the same mechanism—the interruption of cell volume changes during cell migration. This was confirmed by comparing the efficacy of cell migration inhibition to that of NFA, a Cl^−^ ion channel inhibitor, which similarly inhibits cancer cell migration, through the inhibition of cellular volume changes [55]. Data showed no significant difference in anti-migratory efficacy between both treatments.

To the best of our knowledge, there is no prior evidence showing a correlation between nanoparticle uptake, cell volume plasticity, and cell migration. We hypothesised that the degradation product of pSiNPs might play a role in inhibition of cell migration. Under physiological conditions pSiNPs undergo hydrolysis into silicon dioxide, then orthosilicic acid [11]. However, WK1 cells treated with orthosilicic acid showed no significant change in migration across 3 µm pores compared to untreated controls, indicating that the previously observed cell migration inhibition was due to Tf@pSiNPs themselves and not their degradation product. In agreement with our finding, Quignard et al*.* [56] reported that silicon dioxide nanoparticles and orthosilicic acid did not reduce in vitro wound healing and migration of fibroblasts. Since transferrin alone does not significantly inhibit glioma cell migration [10], nor does orthosilicic acid, we are convinced that the anti-migratory effect of Tf@pSiNPs was unlikely a result of its degradation products.

AQPs facilitate intracellular water efflux and are therefore closely related to cell volume plasticity [30]. In particular, AQP9 is overexpressed in gliomas [32]. The function of AQP9 has been shown to be important for cell migration [57] and silicon ion transfer [33]. This prompted investigations into its possible involvement of AQP9 in the cell migration inhibition induced by Tf@pSiNPs. Through a series of AQP9 silencing and cell migration experiments, we observed that the function of AQP9 neither dictated WK1 cell migration across the perforations, nor affected the sensitivity of WK1 cells’ CMI to Tf@pSiNPs. This indicates that the function of AQP9 in WK1 cells are dissimilar to other cell-types, whose migration was inhibited when AQP9 was silenced. For instance, Chen et al*.* [58] found that AQP9 silencing reduced the invasion of prostate cancer, through downregulation of MMP9 and suppression of extracellular signal-regulated kinase (ERK) 1/2 phosphorylation. Lv et al*.* [57] silenced AQP9 in astrocytoma which inhibited RAC serine/threonine‐protein kinase (AKT) activation, hence reducing cell migration and invasion. There was no significant difference in cell migration between AQP9 + and AQP9- cells following treatment with Tf@pSiNPs, suggesting that AQP9 is not involved in the mechanism of action of Tf@pSiNPs. Despite the absence of functional involvement, the AQP9 expression pattern still correlated distinctively with migration. Specifically, AQP9 was localised around the nucleus in non-migrating cells, whereas migrating cells had more diffuse AQP9 expression. This is consistent with Karlsson et al*.* [59], who reported that AQP9 was localised in the nuclear and plasma membranes, and accumulates at the leading edge of the cell when stimulated by a chemoattractant. Such translocation is mediated by AQP9 phosphorylation, under control of the Rac family of GTPases [59]. However, in our study AQP9 silencing did not have a significant effect on cell migration across 3 µm transwells, this may be due to compensation by other AQPs, which are also largely involved in the cytosol volume changes that mediate cell migration [60].

Although AQP9 was not involved in the inhibition of glioma cell migration mediated by Tf@pSiNPs, AQPs have been established as important mediators of cell volume regulation [30]. Therefore, this does not rule out the possibility of AQP involvement as a whole as other AQP subtypes—such as AQP4—which mediates cell invasion [61], may be involved. Yao et al*.* [62] found that the transcriptional factor activator of the hedgehog pathway, Gli, was co-expressed with AQP1 in gliomas, and its activation increased AQP1 expression and hence, glioma cell migration. Dong et al*.* [63] found that AQP8 downregulation reduced glioma cell migration/invasion through cell cycle-inhibition. Yang et al*.* [64] silenced AQP5 and investigated the effect on glioma cell migration, this resulted in cell migration inhibition mediated through the suppression of the epidermal growth factor receptor (EGFR)/ERK/p38 mitogen‑activated protein kinase (MAPK) signalling pathway. Whilst the involvement of other AQP subtypes in the inhibition of cell migration mediated by Tf@pSiNPs has not been eliminated, the lack of AQP9 involvement means that there might be another independent route to attenuate cell volume regulation. Thus, warranting future studies to investigate such interplay.

Based on our findings, we believe that Tf@pSiNPs act by inhibiting the cytosol volume reduction needed to traverse narrow confinements. During cancer cell migration, these cell volume changes are mediated by AQPs and chloride channels [65]. Therefore, the inhibition of either AQPs or chloride channels by Tf@pSiNPs are both plausible mechanisms of action. However, we found that AQP9 is unlikely to be involved in the action of Tf@pSiNPs. Indeed, the internalisation of Tf@pSiNPs may directly interfere with cell volume, without the involvement of AQPs, by modulating the molecular crowding of the cytosol. A recent study on breast cancer invasion described how the degree of intracellular molecular crowding, a pure physical mechanism, could explain cancer cell invasion and disease progression [66]. The link between cell volume and molecular crowding has also been implicated in stem cell research [67]. Importantly, the use of nanoparticles has been suggested as a way to modulate the molecular crowding effect in a biological system [68]. These studies collectively illustrate that internalised Tf@pSiNPs can possibly attenuate cell volume regulation directly by modulating the degree of intracellular molecular crowding. We believe that this approach should be of interest to both nanoparticle scientists and researchers interested in molecular crowding, and that their collaboration on cancer invasion studies could further establish novel ways to manage metastasis.

There is a hierarchal organisation of cancer cells, of which cancer-stem cells have been implicated in driving cancer progression [21], with the expression of CSC markers, such as nestin, being correlated with glioma malignancy [69]. CSCs are responsible for tumour maintenance, treatment resistance and hence cancer recurrence [70, 71]. Therefore, in order to further understand the efficacy of Tf@pSiNPs over the heterogeneity of primary glioma cells, we narrowed our focus onto CSC subpopulations within the heterogeneous WK1 cells. When compared to cells that did not migrate following treatment with Tf@pSiNPs, a larger proportion of migrated cells, which were less susceptible to Tf@pSiNPs, were nestin- and FGFR1-positive, as compared to migration-inhibited cells. Nestin, a class VI intermediate filament protein [27], is a marker for glioma stem cells [26]. Nestin has been found to be involved in cell migration, as it is localised in the leading edge of the tumour [72]. Increased migration of nestin expressing cells is consistent with the literature [73]. Intermediate filaments direct the movement of cells via control of the distribution of the forces in the migrating cells, through control of the acto-myosin network [74]. Ishiwata et al*.* [75] found that nestin silencing reduced glioma migration and invasion. FGFR1 has been found to maintain the stem cell characteristics of cancer cells, through activation of MEK (mitogen-activated protein kinase kinase)/ERK pathways [76] and forkhead box protein M1 (FOXM1) pathway [77]. It is also known that FGFR1 enhances cell migration and proliferation, through activation of the Akt/MAPK pathways and Rho GTPases Rac1/CDC42, respectively [78, 79]. FGFR1 expression has been associated with the increased motility of multiple cancers, for example colorectal [80] and lung [81] cancers, as well as gliomas cells [82]. These generally align with our observation that those nestin / FGFR1 positive subpopulation were more motile even after treatment with Tf@pSiNPs.

Considering that nestin + and FGFR1 + are the phenotypes of glioma CSCs, we believe that the CSC-subpopulation of glioma cells is likely less susceptible to Tf@pSiNPs. An obvious optimisation direction for Tf@pSiNPs would be via functionalisation with CSC-targeting moieties to further suppress the migration of CSC. For example, fibroblast growth factor [28], or ‘AQYLNPs’—a peptide developed by Beck et al*.* [83] found to target nestin-positive cells both in vitro and in vivo. pSiNPs functionalised with transferrin and CSC-targeting moieties might allow for broader migratory inhibition of the different glioma cell subpopulations. However, there is increasing evidence that the characterisation of glioma CSCs is never straightforward, and the plasticity of CSC phenotypes has been recently suggested [84]. More extensive characterisation of the subpopulation is thus essential for further improvement of Tf@pSiNPs anti-migratory approach. It is also important to note that these nanoparticles could potentially be used in combination with conventional chemotherapies, as we have shown in our previous study [18], allowing for a more profound cytotoxic and anti-migratory effect on GBM.

All in all, this study highlights the therapeutic potential Tf@pSiNPs as an anti-metastatic agent. Since Tf@pSiNPs are reported to be able to transverse the BBB [18, 19], they can potentially be administered systemically and orthotopically. We envisage that the simplicity of treatment with Tf@pSiNPs can be compatible with conventional radiotherapy and chemotherapy, where this additional approach can discourage the metastasis of remaining cancer cells, via inhibition of cell migration. Together with all the advantages of pSiNPs such as biocompatibility and scalability, we believe that such an approach will help prevent glioma-recurrence, and thus improve survivability of the disease.

## Conclusion

In this study, we evaluated the effect of Tf@pSiNPs on the migration of primary, patient derived GBM cells (WK1), and dissected its mechanism of inhibition. We observed that Tf@pSiNPs significantly inhibited WK1 cell migration through the interruption of cellular-volume regulation. We demonstrated that the AQP9 expression pattern in WK1 cells correlated with cell migration. However, AQP9 silencing did not affect cell migration across narrow confinements nor did it affect the function of Tf@pSiNPs. GBM cell heterogeneity is the major cause of drug inefficacy. We observed that the majority of cells that were less susceptible to Tf@pSiNPs were nestin and FGFR1 positive, indicating their CSC origin. This highlights that enabling pSiNPs to target CSCs may be a possible optimisation to enhance the efficacy of this approach. These encouraging findings highlight the unique therapeutic potentials of Tf@pSiNPs’ anti-migratory approach in inhibiting local metastasis of glioma and disease recurrence.

## Materials and methods

### Cell culture

Patient-derived primary glioblastoma WKI cells were obtained from the publicly available GBM Q-Cell resource, QIMR Berghofer, Australia (https://www.qimrberghofer.edu.au/commercial-collaborations/partner-with-us/q-cell/) [34, 35, 43]. Cells tested negative for mycoplasma contamination using a PlasmoTest Mycoplasma Detection Kit (ThermoFisher, M7006). Cells were cultured in knockout Dulbecco’s modified Eagle’s medium (K/O DMEM, Gibco, 12660-012), supplemented with GlutaMAX (Gibco, 35050-061), StemPro Neural Supplement (Gibco, A10508-01), and penicillin/streptomycin (Gibco, 15140-122). To help maintain the primary tumour phenotype and genotype, the media was supplemented with 0.1 mg/mL recombinant human Epidermal Growth Factor (Gibco, PHG0314) and 0.05 mg/mL recombinant human Fibroblast Growth Factor basic (Gibco, PHG0024) [72]. Cells were incubated at 37 °C and 5% CO_2_ in a humidified incubator and were passaged at approximately 90% confluency by incubating in acutase solution (Sigma-Aldrich, A694) for 5 min, followed by acutase inactivation. To maintain stem-cell characteristics, cells were only used between passages 16 and 25.

### Cell migration assay

Cell migration assays were carried out using transwell inserts with pore sizes of 3 μm (polyester membrane, Sigma-Aldrich, CLS3472) or 8 μm (polycarbonate membrane, Sigma-Aldrich, CLS3422), in 24 well plates, as described by Justus et al*.* [85].

Cells were seeded in the top chamber at a density of 1 × 10^6^ cells/mL and incubated for 4 h, at 37 °C and 5% CO_2_ in a humidified incubator, to allow cell adhesion and spreading. After attachment, cells were supplemented with new culture media, with or without treatment. Culture media with 10% heat inactivated foetal bovine serum (FBS, Invitrogen, 10,099,141) was added to the bottom well to create a chemotactic gradient. Cells were incubated in a humidified incubator at 37 °C and 5% CO_2_. The chemotactic gradient was renewed every 24 h via addition of new media in both wells. To assess untreated WK1 cell migration over a period of 72 h, separate transwells were run in parallel and fixed at 24, 48 and 72 h, respectively.

The migration of cells, treated with culture media containing 0.1 mg/mL Tf@pSiNPs, 50 μM NFA (Sigma Aldrich, N0630), or 2 mM orthosilicic acid [33] was characterised using the cell migration assay described.

### Porous silicon nanoparticle preparation

Electrochemical anodisation of monocrystalline boron-doped p^+^ silicon wafers (0.01–0.02 Ω cm resistivity) was utilised, as before [10, 18]. The wafers were anodised in a 1:1 (v/v) solution of 38% HF and ethanol. The surface of the wafer was etched with pulses of alternating low and high currents. The produced multilayer was removed from the substrate by increasing the etching current to the electropolishing regions. After drying the films, oxygen and water were removed by placing them under nitrogen flow for 30 min at room temperature. Acetylene flow was then added for a further 15 min. Following this, the temperature was increased to 500˚C and the nitrogen/acetylene flow was maintained. The films obtained were cooled down to room temperature under nitrogen flow, then immersed into undecylenic acid at 120 °C for 16 h. Ball milling, in a 10% (v/v) mixture of undecylenic acid and decane, of the resulting carboxyl-functionalised films produced nanoparticles. These nanoparticles were washed in ethanol and centrifuged to select the desired particle size, and stored suspended in ethanol at 4 °C.

To functionalise with transferrin, 5 mg of the produced nanoparticles, dispersed in ethanol, were centrifuged at 20,000 RCF for 15 min, and then resuspended in 0.1 M 2-(N- morpholino)ethanesulfonic acid (MES) hydrate buffer (Sigma Aldrich, M8250). This was followed by addition of 1-ethyl-3-(3- dimethylaminopropyl)carbodiimide (EDC) hydrochloride (Sigma Aldrich, 03459), sulfo-N-hydroxysulfosuccinimide (sulfo-NHS, Sigma Aldrich, 56485), producing final concentrations of 2.6 mM and 5 mM, respectively. Following a 15 min reaction at room temperature, MES was replaced with phosphate buffered saline (PBS, Gibco, 70011-044), the resulting suspension was added, dropwise, to 10 mg/mL human holo-transferrin (Sigma-Aldrich, T4132), and mixed for 2 h at room temperature, then quenched with 50 mM Tris for 15 min. The resulting Tf@pSiNPs were washed thrice with PBS, centrifuged and stored at 4 °C.

### DLS and zeta potential measurement

The nanoparticles were washed and suspended in PBS, then sonicated for 2 min for measurement of the hydrodynamic size and ζ-potential of the nanoparticles. The Zetasizer Nano ZS (Malvern, UK) was used at 25 °C and a scattering angle of θ = 17°. Each measurement was the average of 13 measurements.

### Transmission electron microscope imaging of nanoparticles

Cyro-transmission electron microscope (TEM) imaging was conducted as described before [10, 18]. 3 µL of Tf@pSiNPs in PBS were dispensed onto a glow discharged copper grid (300 mesh) with lacey carbon film coating (ProSciTech, Australia). This grid was blotted against Whatman 541 filter paper and emerged into liquid ethane using a plunge freezing device at 80% humidity. These were observed under the TEM (FEI, Netherlands), at 120 kV using a Gatan 626 cryo-holder (Gatan, USA), and a low electron dose of 8–10 electrons/Å^2^. FEI Eagle 4kx4k CCD camera (FEI, Netherlands) and AnalySIS v3.2 (Olympus) were utilised for imaging.

### Cellular Si content measured via ICPMS

Glioma cells were seeded onto 12-well plates at a density of 1 × 10^5^ cells per well and incubated at 37 °C and 5% CO_2_ in a humidified incubator. At approximately 90% confluency, cells were washed with warm PBS thrice, and then fresh medium or medium with nanoparticles was added after the final wash (final particle concentration of 50 µg/mL and final volume of 800 µL). After a 1 h incubation, the cells were washed with warm PBS twice to remove unbound nanoparticles. Cells were then detached with trypsin/ethylenediamine tetraacetic acid (0.05%, ThermoFisher, Cat.25300062), resuspend in 100 µL of 1% Bovine Serum Albumin (BSA)/PBS solution, and kept in the fridge or ice until analysis. The elemental analysis was performed using Agilent 8900 ICP-QQQ-MS fitted with an inert PFA sample introduction system. Si was measured in MS/MS mode using Hydrogen reaction gas on mass at m/z 28. 57 µL of the cell samples were digested in 1 M NaOH at a ratio of 1:3 (w/w) overnight at room temperature. The solutions were further diluted with MilliQ water and submitted for elemental analysis. The quantification was performed by external calibration with Si standards for inductively coupled plasma, which were prepared in 0.1 M NaOH from a 1000 mg/L Si standard (HPS standards), under identical settings.

### AQP9 silencing

WK1 cells were seeded in transwells as previously described. Cells were incubated for 4 h, at 37 °C and 5% CO_2_, to allow cell adhesion and spreading, cells were then washed twice with PBS, followed by transfection with AQP9 siRNA (Santa Cruz, sc-42371). Lipofectamine RNAiMAX (Invitrogen, 13,778-075) and AQP9 siRNA were separately diluted 3:50 and 1:50, respectively, in Opti-Minimal Essential Medium (Opti-MEM) Reduced Serum Medium (Gibco, 31985070). The solutions were mixed in a 1:1 ratio and incubated at room temperature for 5 min. The mixture was then diluted 1:10 in Opti-MEM, before addition to cells. Cells were incubated for 15 h at 37 °C and 5% CO_2_, after which transfection media was replaced with fresh culture media. After a further 24 h incubation, cells were further processed for follow-up assays.

### Cell staining

To identify and image cells on the transwell insert, the perforated insert membranes were excised and immersed in PBS. Following three washes with PBS, cells were fixed with 4% paraformaldehyde (PFA, Sigma-Aldrich, P6148) in PBS for 10 min at room temperature. Cells were washed twice and were then permeabilised with 0.1% Triton X-100 in PBS for 5 min at room temperature, followed by three more washes with PBS. Cells were incubated with 5 mg/mL rhodamine-phalloidin (Invitrogen, R415) and 10 mg/mL Hoechst 33,342 (Sigma Aldrich, B2261) diluted 1:500 and 1:5000 in PBS, respectively, in the dark for 1 h. Cells were then washed with PBS twice and mounted between two coverslips with ProLong Diamond Antifade Mountant (Invitrogen, 1,916,897).

Cells for immunofluorescence microscopy imaging were washed, fixed and permeabilised as aforementioned. Cells were then blocked in 1% BSA (Gibco, 37525) in PBS for 30 min. An appropriate primary antibody—nestin antibody (Novus, 4D11) or FGFR1 antibody (Novus, M19B2) diluted 1:250 or 1:100, respectively, in 1% BSA, was added to the cells and incubated overnight at 4 °C. After three washes, F(ab’) goat anti-mouse Alexa 488 (Invitrogen, A11017) diluted 1:1000 in 1% BSA, with 1:500 rhodamine-phalloidin, was added and incubated with cells for an hour in dark at room temperature. Cells were then washed three times with PBS. Nuclei were stained with Hoechst diluted 1:5000 in PBS and left for 20 min. Cells were washed in PBS three times and mounted using ProLong between two coverslips.

To identify if Tf@pSiNPs cause any changes in the expression of AQP9, WK1 cells were seeded into the wells of a Lab-Tek II Chamber slide (ThermoFisher, 154534) at a seeding density of 5 × 10^4^ cells/cm^2^ and incubated at 37 °C and 5% CO_2_ in a humidified incubator. At approximately 70% confluency, cells were treated with 0.1 mg/mL Tf@pSiNPs and incubated for a further 48 h. Cells were then fixed and stained as described above, with AQP9 antibody (G-3, Santa Cruz, 74409) used as the primary antibody, diluted 1:150 in 1% BSA.

To identify if AQP9 affects the uptake of Tf@pSiNPs, cells were seeded into the 8 wells of a Chamber slide as described before. Control cells and AQP9 siRNA-transfected cells were then exposed to 0.1 mg/mL Cyanine5-labelled Tf@pSiNPs and incubated for 48 h. After incubation, the cells were washed and fixed as described above and washed three times with PBS at room temperature. Cells were then incubated with Hoechst diluted 1:5000 in PBS for 25 min and washed three times with PBS. Vybrant CM-DiI (chloromethylbenzamido-1,1′-dioctadecyl-3,3,3′,3′-tetramethylindocarbocyanine perchlorate) cell-labelling solution membrane staining dye (Invitrogen, V22888) diluted 1:200 in Dulbecco’s PBS (Gibco, 1410-144) was then added to the cells, and incubated at 37 °C for 4.5 min, then at 4 °C for 15 min. Cells were then washed with PBS three times and incubated at 37 °C for 10 min with every wash. Finally, cells were fixed by addition of 4% PFA for 10 min, at 37 °C and washing three times with PBS.

### Imaging and analysis

Imaging was carried out using Leica SP8 Lightning Confocal Microscope (Leica Microsystems, Germany). Since transwell membranes are not flat, Z-stack images were taken and maximum projection was used to visualise all cells on a single image. Each side of the membrane was imaged at 5 random locations. Nuclei were counted using ImageJ [86]. The CMI was calculated using the equation:

$${\text{CMI}}\left( \% \right) = \frac{{{\text{Number}}\;{\text{of}}\;{\mkern 1mu} {\text{cells}}\;{\mkern 1mu} {\text{on}}\;{\mkern 1mu} {\text{the}}\;{\mkern 1mu} {\text{underside}}\;{\mkern 1mu} {\text{of}}\;{\mkern 1mu} {\text{the}}{\mkern 1mu} \;{\text{membrane}}}}{{{\text{Number}}\;{\mkern 1mu} {\text{of}}{\mkern 1mu} \;{\text{cells}}{\mkern 1mu} \;{\text{on}}{\mkern 1mu} \;{\text{both}}\;{\mkern 1mu} {\text{sides}}{\mkern 1mu} \;{\text{of}}\;{\mkern 1mu} {\text{the}}\;{\mkern 1mu} {\text{membrane}}}} \times 100$$

To identify nestin and FGFR1 expression, the area stained by the antibodies was measured using ImageJ [86]. This was then divided by the total number of nuclei observed. The expression on either side of the membrane was compared using the equation:

$$\mathrm{Expression ratio}= \frac{\mathrm{Expression\, on\, the\, underside\, of\, the\, membrane}}{\mathrm{Expression \,on \,the \,topside \,of \,the \,membrane}}$$

Cell proliferation rate was quantified as the ratio between the number of cells 24 and 48 h post-seeding using the equation:

$$\mathrm{Cell \,proliferation \,rate}= \frac{\mathrm{Number \,of \,cells \,at }48\mathrm{ \,h}}{\mathrm{Number \,of \,cells \,at }24\mathrm{ \,h}}$$

All experiments were performed in triplicate. Data is presented as mean ± standard deviation (SD). The Student's t-test or one-way analysis of variance (ANOVA) were used to assess the significance of the data. The alternative hypothesis was accepted at the 95% significance level (p < 0.05). Statistical analyses were conducted using GraphPad Prism 8 (San Diego).

## Data Availability

The datasets used and/or analysed during the current study are available from the corresponding author on reasonable request.
